# Training and capacity building: Developing a support framework for researchers using administrative data

**DOI:** 10.23889/ijpds.v9i5.2565

**Published:** 2024-09-18

**Authors:** Emily Oliver, Saba Mir, Emma Gordon

**Affiliations:** 1 ADR UK

## Background

Advances in data infrastructure and data access legislation supporting the safe use of securely held administrative data have led to increased data being available for research in recent years. While uptake by researchers has increased, opportunities for innovative, impactful research in the public interest outweigh the number of researchers using these data.

## Objective

To drive up use, Administrative Data Research UK (ADR UK) embarked on the delivery of a Training and Capacity Building Strategy. Its objectives are to raise awareness across disciplines; to support more researchers to use administrative data for the first time; and to keep them using it for more advanced research, using a comprehensive researcher support framework.

## Approach

By mapping potential users of administrative data across multiple career stages, we identified activities, products and approaches to test. These range from low-cost, high-reach activities to in-depth, targeted approaches, and include sustainable products to ensure longevity.

## Results

Testing and evaluation of these varied approaches has enabled us to understand more about administrative data users and their needs, individually and collectively. They have underlined the need for sustained awareness and development of available data and opportunities, supported by accessible tools and resources to maintain momentum in what can be a frustrating research environment.

## Conclusions and Implications

The framework we've created offers scaffolding from which to hang multiple approaches to drive up the use of administrative data. Communities and networks play an important role in supporting users in this niche area of study across different sectors and disciplines, and in creating a sustainable future for research in this field.

